# Overshoot of the Respiratory Exchange Ratio during Recovery from Maximal Exercise Testing in Young Patients with Congenital Heart Disease

**DOI:** 10.3390/children10030521

**Published:** 2023-03-07

**Authors:** Marco Vecchiato, Andrea Ermolao, Emanuele Zanardo, Francesca Battista, Giacomo Ruvoletto, Stefano Palermi, Giulia Quinto, Gino Degano, Andrea Gasperetti, Massimo A. Padalino, Giovanni Di Salvo, Daniel Neunhaeuserer

**Affiliations:** 1 Sports and Exercise Medicine Division, Department of Medicine, University of Padova, 35128 Padova, Italy; 2 Clinical Network of Sports and Exercise Medicine of the Veneto Region, 35131 Padova, Italy; 3 Public Health Department, University of Naples Federico II, 80131 Naples, Italy; 4 Pediatric and Congenital Cardiac Surgery Unit, Department of Cardiac, Thoracic, Vascular Sciences and Public Health, University of Padova, 35128 Padova, Italy; 5 Pediatric and Congenital Cardiology Unit, Department for Women’s and Children’s Health, University of Padova, 35128 Padova, Italy

**Keywords:** cardiopulmonary exercise test, CHD, RER, Fontan, Fallot, coarctation, transposition, functional evaluation

## Abstract

Introduction: The overshoot of the respiratory exchange ratio (RER) after exercise is reduced in patients with heart failure. Aim: The present study aimed to investigate the presence of this phenomenon in young patients with congenital heart disease (CHD), who generally present reduced cardiorespiratory fitness. Methods: In this retrospective study, patients with CHD underwent a maximal cardiopulmonary exercise testing (CPET) assessing the RER recovery parameters: the RER at peak exercise, the maximum RER value reached during recovery, the magnitude of the RER overshoot and the linear slope of the RER increase after the end of the exercise. Results: In total, 117 patients were included in this study. Of these, there were 24 healthy age-matched control subjects and 93 young patients with CHD (transposition of great arteries, Fontan procedure, aortic coarctation and tetralogy of Fallot). All patients presented a RER overshoot during recovery. Patients with CHD showed reduced aerobic capacity and cardiorespiratory efficiency during exercise, as well as a lower RER overshoot when compared to controls. RER magnitude was higher in the controls and patients with aortic coarctation when compared to those with transposition of great arteries, previous Fontan procedure, and tetralogy of Fallot. The RER magnitude was found to be correlated with the most relevant cardiorespiratory fitness and efficiency indices. Conclusions: The present study proposes new recovery indices for functional evaluation in patients with CHD. Thus, the RER recovery overshoots analysis should be part of routine CPET evaluation to further improve prognostic risk stratifications in patients with CHD.

## 1. Introduction

Congenital heart disease (CHD) accounts for nearly one-third of all major congenital anomalies and its birth prevalence worldwide is suggested to vary [1]. Recent data extracted from the European Surveillance of Congenital Anomalies estimated the average total prevalence of CHD in Europe is around 8.0 per 1000 births [2]. In this patient population, long-term survival is decreased [3], with lesion severity and repair status as major risk factors for excess mortality [4]. However, due to improvements in medical, surgical, and intensive care interventions, the life expectancy of patients born with CHD has been rising over time [3].

Cardiorespiratory fitness was found to be highly heterogeneous both within and between individuals with CHD diagnoses [5]. In this context, cardiopulmonary exercise testing (CPET) has emerged as an important tool for risk stratification and may guide clinicians in assessing prognosis and planning interventions in CHD patients [6,7]. CPET may also be useful for diagnostic purposes as well as for decision-making making.

Most of the studies about CPET have focused on the cardiopulmonary response during exercise; however, there is less evidence about the respiratory gas indices during recovery with most studies focusing only on oxygen uptake (VO_2_) kinetics [8,9]. Most studies on the recovery phase of patients with heart disease are on adult patients with heart failure (HF) [10,11,12]. These studies demonstrated that patients with HF exhibit an increased VO_2_ delay during the recovery phase after maximal CPET compared to controls and this delay is associated with the severity of diseases [13]. Recently, the transient increase, defined as overshoot, of some CPET parameters during the recovery phase, such as the respiratory exchange ratio (RER), has aroused scientific and clinical interest [12]. The magnitudes of these parameters have been compared between HF patients and healthy subjects, demonstrating that overshoots tended to be more pronounced in subjects with better cardiopulmonary function during exercise [12].

Cardiovascular recovery after exercise appears to be faster in children than in adults [14,15], but data about the recovery after CPET in young patients with CHD is limited. VO_2_ recovery kinetics and heart rate (HR) recovery are prolonged in patients with different types of CHD [16,17] but little is known about the prognostic and diagnostic value of this data and how it can guide clinical decision-making [18,19].

Therefore, the present study aimed to evaluate the behavior of the respiratory gas exchange indices during recovery in young patients with CHD, assessing the impact of different conditions compared with an age-matched healthy control group. Furthermore, it will be discussed how these recovery parameters might be used for prognostic risk stratification in clinical routine. 

## 2. Materials and Methods

### 2.1. Study Design and Population Characteristics

This was a retrospective observational cross-sectional study that included all young patients with CHD (aged between 7 and 20 years) who were evaluated at our Sports and Exercise Medicine Division between 2018 and 2021 for cardiovascular screening/follow-up, sports eligibility assessment, and/or exercise prescription [20]. Patients with different CHD were compared to highlight any functional and prognostic differences between their CPET exercise and recovery parameters. The selected CHD were the 4 most represented within our population: transposition of great arteries (TGA), patients with univentricular CHD who underwent Fontan procedure (Fon), aortic coarctation (CoA), and tetralogy of Fallot (ToF). Other or complex CHD, as well as all patients with beta-blocker therapy and/or pacemaker, were excluded from this study. The other exclusion criteria were related to absolute contraindications to CPET evaluation, as well as musculoskeletal disease that would impede maximal exercise testing. Moreover, tests with gas monitoring of fewer than four minutes during the recovery phase were excluded.

A control group of apparently healthy subjects was added, consisting of children who were referred for pre-participation screening or for minor complaints during exercise, such as chest pain, palpitations or breathing difficulties, but were declared negative after the diagnostic process. The control group was selected to match the patient study population with regard to gender, age, and body mass index (BMI).

In accordance with legal regulations, the Code of Medical Ethics and the Declaration of Helsinki, subjects were duly informed of the risks, benefits, and stress deriving from the study protocol and signed a written informed consent form. This study was approved by the local Ethics Committee for Clinical Research (protocol code 302n/AO/22).

### 2.2. Cardiopulmonary Exercise Testing

For each patient, personal history was collected and a physical examination was conducted. Each subject underwent a standardized, incremental, maximal 12-leads ECG-monitored CPET (Masterscreen CPX system Jaeger, Carefusion, Hoechberg, Germany) using a treadmill (T170 DE-med, h/p/cosmos, Nussdorf-Traunstein, Germany) until a rating of perceived exertion (RPE) ≥ 18/20 of Borg Scale was reached and metabolic, cardiovascular, or ventilatory signs of exhaustion appeared. Blood pressure was measured both at rest and during CPET and its recovery phase. The age-predicted heart rate (HR) was calculated with the following formula: (220-age) bpm. The respiratory gas exchange (VO_2_, VCO_2_) and ventilation (VE) were monitored through the breath-by-breath mode and at least until the fourth minute of recovery. The first ventilatory threshold (VT) was identified through the V-slope method. When the VT was not clearly identifiable, it was determined by the consensus of two physicians within the following group (A.E., G.D., A.G., or D.N.). The respiratory compensation point (RCP) was determined with the same principle considering the ventilatory equivalents and the partial pressure of end-tidal carbon dioxide (PETCO_2_). The VE/VCO_2_ slope was calculated as the coefficient of linear regression from the beginning of the exercise (removing possible initial hyperventilation) to the RCP. The oxygen uptake efficiency slope (OUES) was determined by the slope of the regression line between VO_2_ and the logarithm of VE [21,22].

### 2.3. Overshoot Analyses

**Figure 1:**
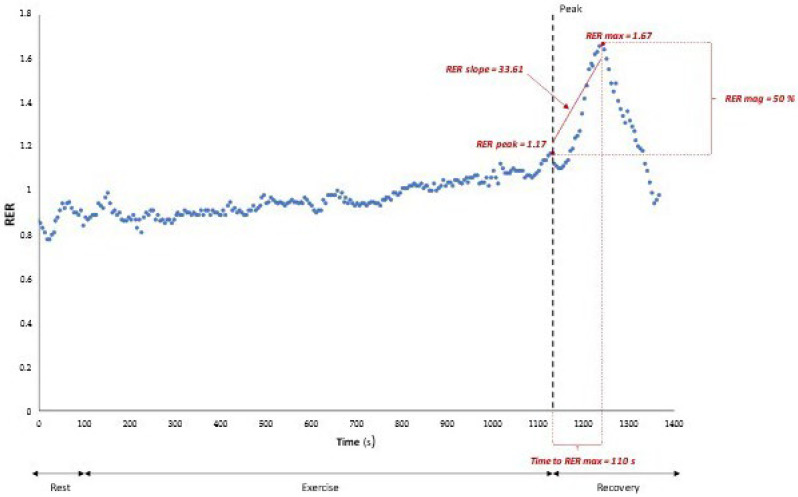
The respiratory exchange ratio (RER) recovery parameters in a healthy subject.

The RER overshoot was analyzed by assessing five parameters (Figure 1):

- the RER at peak exercise (RER peak) was defined as the highest value of the RER reached during exercise;
- the highest RER value reached during the recovery phase (RER max);
- the difference between RER max and RER peak, calculated as the percentual increase during the recovery phase; i.e., RER magnitude (RER mag);
- the slope of the RER calculated by linearly regressing the RER data between RER peak and RER max during the recovery phase (RER slope);
- the time between RER peak and RER max (Time to RER max) [12].

### 2.4. Ventricular Function Assessment

Furthermore, all subjects were assessed to obtain data regarding ventricular systolic function. Data regarding ventricular systolic function were obtained by echocardiographic evaluations, which have been performed in the context of the routine follow-up of these patients at the Department of Women’s and Children’s Health of University of Padova. The parameter chosen to quantify the systolic function of the left ventricle is the ejection fraction (LVEF), which indicates the ratio (expressed as a percentage) between the volume of blood expelled during systole from the left ventricle and the end-diastolic volume. For the systolic function of the right ventricle, on the other hand, tricuspid annular plane systolic excursion (TAPSE), which is the displacement of the tricuspid valve plane towards the cardiac apex during ventricular systole, as well as the fractional area change (FAC), as shortening percentage of the right ventricle between systole and diastole, were evaluated.

### 2.5. Statistical Analyses

Data are expressed as a mean ± the standard deviation. The normality was assessed using the Shapiro–Wilk test. *T*-tests for the normally distributed variables and Mann–Whitney U tests for the non-normally distributed variables were used. The various classes of CHD were compared with each other and with controls by an ANOVA test for normally distributed variables and with a non-parametric test for non-normally distributed variables. Patients with CHD were further classified to investigate the RER recovery parameters in subgroups with potential prognostic differences. Patients were grouped according to the VE/VCO_2_ slope into ventilatory classes: I (VE/VCO_2_ slope < 30), II (VE/VCO_2_ slope between 30 and 35.9), and III (VE/VCO_2_ slope between 36 and 44.9); no patients belonged to ventilatory class IV (VE/VCO_2_ slope > 45). The correlations were evaluated with Pearson’s index for normally distributed variables and with Spearman’s index for non-normally distributed variables. The statistical analyses were executed with IBM SPSS Statics software version 26. A statistical significance level of *p* ≤ 0.05 was applied.

## 3. Results

### 3.1. Patients Selection

In total, 131 young subjects were initially recruited for the aim of the study, including 103 patients with CHD and 28 control subjects. In the CHD group, seven patients were excluded from the study because it was not possible to clearly identify an RER max during the time recorded; one patient was excluded due to a sampling error, and two patients were excluded because they did not reach the criteria for metabolic exhaustion. Therefore, the CHD study group was made up of 93 subjects: 23 TGA, 22 Fon, 24 CoA, and 24 ToF. All patients with TGA correction underwent the arterial switch procedure. Moreover, 18 patients of the Fontan group presented a left dominant ventricle, whereas 7 had a right dominant ventricle and 1 patient underwent a staged biventricular conversion. The degree of pulmonary valve regurgitation and right ventricle outflow tract stenosis was rather heterogeneous in the ToF group. In the control group, three patients were excluded because it was not possible to clearly identify a RER max during the recovery phase, and one patient because he was unable to reach the needed metabolic criteria for exhaustion. The control group, therefore, was composed of 24 subjects.

### 3.2. Baseline Characteristics

The general anthropometric and clinical characteristics of the study participants are represented in Table 1.

Resting systolic and diastolic blood pressures were higher in CHD patients than in healthy controls (*p* = 0.022 and *p* < 0.001, respectively). Moreover, statistically significant differences were found between the 4 subgroups of CHD in systolic blood pressure (*p* = 0.001), with patients with CoA having the highest mean resting SBP (119.92 ± 14.65 mmHg). None of the subjects included in the control group had a low blood oxygen saturation at rest, while five patients with CHD (all belonging to the Fon group) desaturated at rest before the exercise phase.

### 3.3. Cardiopulmonary Exercise Testing and Echocardiographic Assessments

All patients performed their respective maximal CPET with the same protocol (Bruce Ramp) reaching a RPE ≥ 18/20 on the Borg scale, with no reported symptoms. The results of the CPET and the main indices of cardiac contractility analyzed with echocardiography are shown in Table 2.

HR peak was lower in the CHD group than in the control group (*p* = 0.013). The comparison of HR peak between the subgroups was also statistically significant (*p* = 0.001), with patients with Fontan having the lowest median (176 bpm). Even the HR recovery after one minute was lower in patients with CHD compared to controls (*p* = 0.007), with Fon patients showing the slowest recovery. As for the oxygen pulse (O_2_ pulse), an anomalous behavior was recorded in 30% of patients with CHD, whereas all healthy controls had a normal O_2_ pulse behavior during the test. Patients with CHD showed lower values of the O_2_ pulse in terms of percentage of predicted, when compared to healthy controls (*p* = 0.005) with still Fon patients presenting lower values compared to the other three CHD subgroups. The analysis of peripheral saturation at peak exercise (SpO_2_ peak) showed that 19 patients with CHD desaturated at peak exercise (12 from the Fon group), while none of the healthy controls had a lower-than-normal peripheral saturation.

Pairwise comparisons between the various parameters are shown in Supplementary Table S1. Most significant differences have been found between the Fontan group, which had the greatest functional impairment, and the CoA and control groups. CoA patients showed higher HR peak and HR peak (%) compared to ToF and Fon patients but similar to TGA patients. Furthermore, aerobic capacity was significantly lower in patients with CHD compared to healthy subjects; statistically significant differences were also displayed between the four subgroups (in both cases *p* < 0.001) with Fon patients recording the lowest VO_2_ peak (32.05 ± 5.90 mL/min/kg; 76.64 ± 14.40% of predicted) and CoA patients with the highest aerobic capacity (40.98 ± 8.40 mL/min/kg; 99.00 ± 17.10% of predicted).

### 3.4. Overshoot Analysis

**Figure 2:**
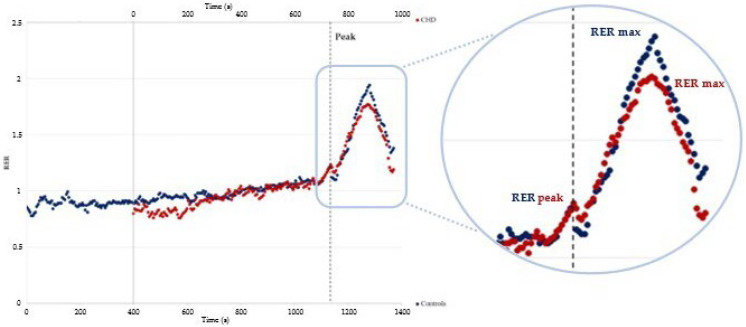
Example of two patients (a healthy subject in blue and a patient with CHD in red) presenting the same respiratory exchange ratio at peak exercise (RER peak) but different maximal values of respiratory exchange ratio during the recovery phase (RER max).

Table 3 shows the comparison between the parameters concerning the phenomenon of RER overshoot during the recovery phase between study groups. All included patients showed an increase, defined as an overshoot of the RER after exercise. Although during exercise patients with CHD and controls showed a similar RER peak, the behavior of the RER during recovery was significantly different. Moreover, RER max, RER mag, and RER slope revealed a lower RER overshoot for patients with CHD when compared to the healthy controls (Figure 2). However, no statistically significant difference was found for the RER peak, RER max, RER slope and Time to RER max parameters when CHD subgroups were compared. Only the RER mag was higher in the controls and patients with CoA when compared with the Fon, ToF, and TGA groups.

The correlations between RER overshoot parameters during the recovery phase and some of the main cardiorespiratory fitness and efficiency indices were assessed (Table 4).

**Figure 3:**
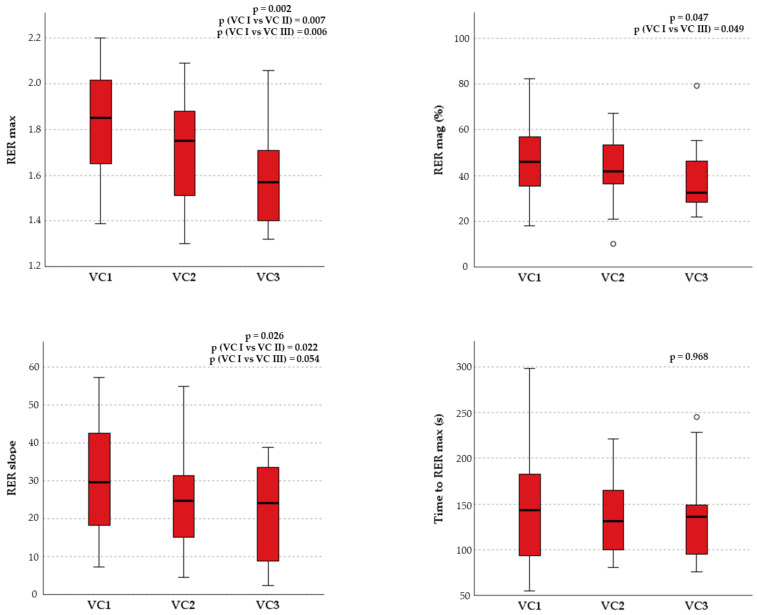
The RER overshoot parameters (RER max, RER mag, RER slope and Time to RER max) between patients of different ventilatory classes. VC= ventilatory class.

HR peak showed significant correlations with both RER max (ρ = 0.323; *p* < 0.001) and RER mag (r = 0.366; *p* < 0.001). A significant negative correlation between RER mag and HR/VO_2_ slope was displayed, as well as a positive correlation between RER max and HRRec after one minute. Although the time to RER max and the RER slope showed slight correlations with the principal CPET parameters, RER max and RER mag were significantly correlated with important cardiorespiratory fitness and efficiency indices, such as VO_2_ peak and OUES. Moreover, when grouped by ventilatory classes, the RER recovery parameters, except for time to RER max, were significantly higher in patients of ventilatory class I compared with patients belonging to ventilatory classes II and III (Figure 3). No statistically significant correlations between the RER recovery parameters and resting echocardiographic data were found, except between RER max and TAPSE.

## 4. Discussion

To the best of the authors’ knowledge, this is the first study evaluating the overshoot parameters of the respiratory gas exchange and specifically the behavior of the RER during recovery from maximal CPET in young patients with CHD. The main results of the present study are the following:

- All patients with CHD presented an overshoot of the RER during recovery after maximal CPET.
- Patients with CHD showed reduced RER recovery overshoot compared to healthy subjects.
- Although there are significant differences regarding the cardiopulmonary response during exercise between the subgroups of CHD, no differences in the RER recovery parameters were evident.
- RER recovery parameters significantly correlated with the most important cardiorespiratory fitness and efficiency indices, independently from the RER peak reached during exercise.

### 4.1. Why Is the CPET Recovery Phase Relevant in Patients with CHD?

Currently, the cardiopulmonary response during exercise has been widely studied in different populations but there is still little evidence of the CPET parameters’ behavior during the recovery phase [8]. Some authors described delayed kinetics of VO_2_ recovery in patients with HF after maximal and submaximal incremental exercise testing compared to healthy subjects [13,23,24], showing that these findings were associated with a worse prognosis in these patients [24,25]. Slow recovery of energy stores in skeletal muscles was deemed to be responsible for the delayed VO_2_ recovery [26]. In addition, in patients with HF, a delayed recovery of VE and VCO_2_ was also found. This phenomenon has been attributed to the retention of CO_2_ in the muscles after exercise, justifying the consequent increase in ventilation to maintain a state of eucapnia [24]. In this regard, it is noteworthy to underline that parameters describing the recovery phase seemed to have a more significant correlation than peak values with muscle strength in both healthy controls and patients with HF [27].

In the latter, the phenomenon of VO_2_ overshoot during the first part of the recovery phase has also been described: it is defined by a further VO_2_ growth compared to the peak values [28]. This overshoot has been found in some cardiac patients and it seems to be associated with a worse prognosis [29]. Other authors also found a paradoxical increase in cardiac output in the recovery phase after CPET [11], which may explain VO_2_ overshoot. This increase in cardiac output would be attributable to a reduction of peripheral vascular resistances at the end of the exercise but also to the contribution of skeletal muscles to repay the oxygen debt or to a relatively slower decline in the blood concentration of catecholamines during recovery [11,30].

More recently, the overshoot phenomenon of gas exchange indices, such as RER and VE/VO_2_ during the recovery phase after maximal CPET, has been described [12]. Takayanagi et al. identified an attenuation of this phenomenon in patients with HF when compared to healthy subjects [12]. The overshoot of gas exchange indices seems to be a direct consequence of VE and VCO_2_ returning to normal more slowly than VO_2_, due to the carbon dioxide deposits produced by the anaerobic metabolism during exercise [12]. 

Some authors reported a delay in gas exchange recovery in patients with different types of CHD, but they mainly focused on the VO_2_ recovery kinetics [16,17]. Since most of the literature concerning the evaluation of CHD by CPET focuses on the exercise phase, this study aimed to analyze the behavior of the main cardiopulmonary indices during recovery in a population of young patients with CHD and to compare it with an age-matched healthy population. The RER was chosen as the most suitable parameter to be evaluated as it reflects simultaneously both the VO_2_ and VCO_2_ trend, with possibly slowed kinetics during the recovery phase [16,17,31].

### 4.2. Exercise Phase

Patients with CHD are subjects who, despite the improvement in medical and surgical therapies that occurred over the last decades, are still forced to live their whole life with the pathophysiological alterations due to their disease and to the sequelae of surgical interventions. These alterations mainly involve the cardiovascular system with consequent functional limitations, but, in complex CHD the whole oxygen transport system might be affected [32,33]. Therefore, a comprehensive functional evaluation with maximal CPET is strongly recommended in current guidelines [34]. Patients with CHD have reduced cardiorespiratory fitness and efficiency during exercise when compared to healthy controls. This is widely demonstrated in the literature and confirms the possible presence of cardiogenic limitations to exercise in patients with CHD [35]. Indeed, children and adults with CHD, in particular after surgical repair, have lower maximal aerobic and functional capacity compared to controls [36,37]. A recent systematic review and meta-analysis investigating children and adolescents with CHD reported a lower exercise capacity and cardiorespiratory efficiency compared with healthy controls as shown by worse VO_2_ peak, maximal power, VE/VCO_2_ slope, O_2_ pulse, and HR max [38].

Moreover, exercise capacity differs significantly across the spectrum of CHD [35]. Simple CHD present usually better exercise parameters compared to complex CHD. In our study, CoA patients had a higher HR peak compared to ToF and Fon patients. Moreover, Fon patients presented mild chronotropic incompetence compared to other CHD subgroups, probably related to an abnormal cardiac filling rather than sinoatrial node dysfunction. In accordance with previous studies, patients with complex CHD, in particular patients with univentricular circulation who underwent a Fontan procedure, presented the lowest VO_2_ peak and highest VE/VCO_2_ slope values [39]. On the other hand, patients with CoA and patients with TGA, in particular those who received an arterial switch operation, were found to have the highest VO_2_ peak and lowest VE/VCO_2_ slope values [40]. Our work confirms this previous evidence with Fon patients presenting the lowest maximal and submaximal cardiorespiratory fitness during exercise. Indeed, a proportion of them showed desaturation at rest and during exercise, with the worst values for aerobic capacity and ventilatory efficiency among the CHD groups investigated. Moreover, despite patients with TGA presenting a lesion with high complexity, restoring correct anatomy and physiology during early life seem to ensure an almost normal exercise capacity and cardiorespiratory fitness [35].

### 4.3. Recovery Phase

The focus of this study was related to the RER recovery parameters after maximal CPET. Patients with CHD presented significantly reduced RER max, RER mag, and RER slope compared to healthy controls, despite the RER peak value during exercise being comparable between the two groups (1.22 ± 0.11 vs. 1.23 ± 0.12). These findings seem to confirm that, in subjects with cardiogenic limitations to physical exercises, such as patients with CHD, the RER overshoot phenomenon appears to be reduced [12]. Although the included chronic conditions do not share the same pathophysiological mechanism, it is possible that the underlying cardiac impairment leading to the reduced RER overshoot of patients with CHD may be similar to what has been described in patients with HF [12].

A delay in VO_2_ recovery kinetics and HR recovery has already been demonstrated in young patients with different CHD [16]. An impaired right-sided hemodynamic and central autonomic nervous activity may lead to a delay in recovery indices [18] with possible implications also for clinical decision-making [19]. Most studies on patients with CHD have focused on adult populations [40]. In one of the few works investigating the recovery phase of a young population with CHD, patients with ToF presented diminished exercise capacity and slower recovery of VO_2_ and VCO_2_ compared to healthy subjects. Those patients with the worst exercise capacity also showed the slowest recovery indices [31]. This delay seemed to correlate to ventricular contractility indices, suggesting the crucial role of ventricular function during the recovery after physical exercise [31].

Interestingly, patients with different classes of CHD did not show significant variances in RER recovery indices. This confirms previous studies on adults showing that gas exchange recovery after exercise testing is prolonged in patients with CHD, independently of the congenital heart lesion [17]. However, comparing our results with prior studies, RER mag of young patients with CHD (44.4 ± 14.8%) was higher compared to older patients with HF, kidney transplant recipients but also healthy older subjects (21.4 ± 12.4%, 28.4 ± 12.7% and 29.3 ± 10%, respectively) [12,41]. These findings suggest that age appears to be a crucial factor in determining this phenomenon in the recovery phase, as subjects with the established cardiac disease show higher values than healthy older subjects, even in CHD with the lowest cardiorespiratory fitness (RER mag in Fon group: 42.31 ± 13.10%).

### 4.4. RER Overshoot and Cardiorespiratory Fitness/Efficiency

The RER recovery indices showed interesting correlations with cardiorespiratory efficiency even in CHD, corroborating that it is possible to implement the CPET evaluation in this clinical population. HR/VO_2_ slope describes the subject’s ability to adequately raise HR to meet the increased metabolic demands during exercise, and it is a cardiocirculatory efficiency index that has been poorly studied in the literature so far, particularly in patients with CHD [42]. A significant negative correlation was found between RER mag and HR/VO_2_ slope (r = −0.232, *p* = 0.004). It could be hypothesized that patients with a hyperkinetic response during exercise and thus lower cardiocirculatory efficiency have also a reduced RER overshoot in the recovery phase, probably due to cardiac limitations. Furthermore, patients with better exercise tolerance and thus higher HR peak have shown a more significant RER overshoot. It needs to be investigated whether this observation is due to cardiac limitations regarding the chronotropic response or simply due to lower exercise tolerance.

The RER mag showed significant correlations with relevant indices of cardiorespiratory fitness and efficiency such as VO_2_ peak and OUES, which were comparable with those previously described for patients with HF [12]. This shows how the overshoot phenomenon is closely related to maximal and submaximal aerobic capacity. Different from previous works, no correlation between RER mag and VE/VCO_2_ slope was found [12,41]. This could be explained by the fact that the population in this study was young and might not have yet developed a relevant degree of ventilatory-perfusion mismatch. Alternatively, since RER peak and RER max seem to negatively correlate with VE/VCO_2_ slope, these data may suggest that ventilatory-perfusion mismatch has a huger impact on exercise tolerance and affects less the recovery phase [43]. To evaluate the potential clinical application of the RER overshoot, patients with CHD were grouped according to their ventilatory classes, which reflect the cardiorespiratory efficiency and a possible ventilation-perfusion mismatch during exercise. Patients with better ventilatory classes showed higher RER recovery overshoots compared with those belonging to worse ventilatory classes, similar to what was reported about kidney transplant recipients [41]. Furthermore, a vigorous RER overshoot seems to be an index of better cardiorespiratory performance and a better prognosis in patients with CHD. This supports the proposal that the analysis of CPET metrics during recovery may provide valid additional information for the test interpretation.

Finally, correlations between RER overshoot and resting biventricular function were investigated. No significant correlations between RER mag and echocardiographic parameters were found, as previously reported between RER mag and LVEF in patients with HF, suggesting the absence of a direct relationship between RER overshoot and ventricular function at rest [12]. However, there is still the need to study how limitations in the response of cardiac output during exercise may influence the CPET overshoot recovery parameters. In this regard, data from invasive and/or non-invasive measurements of cardiac output during exercise could be useful to better understand the direct impact of cardiac limitations on these recovery metrics.

### 4.5. Limitations and Perspectives

This was a retrospective study assessing the recovery phase after maximal CPET based on routinely performed clinical assessments. The sample size was limited because a long-lasting evaluation of the recovery phase with gas exchange data was not routinely performed in our laboratory before January 2018, when a dedicated recovery protocol was created. A larger sample and specific trials are needed to investigate the impact of ventricular function (during rest and exercise) on the RER recovery overshoot, as echocardiographic data have been assessed for clinical purposes and were thus not available for all patients. Moreover, those patients with a peak RER < 1.1 were excluded from the study, for consistency with previous literature and to avoid possible confounding in the assessment of the recovery CPET parameters, especially RER.

The recovery phase after exercise has been poorly explored in pathological populations and often with heterogeneous methodologies. The present study could help to highlight the possibility of incorporating and standardizing variables of the recovery phase in CPET interpretation, aiming to improve the diagnostic and prognostic stratification of these patients. Future trials should analyze the behavior of gas exchange indices after maximal exercise testing in populations with different functional limitations, to improve the understanding of the pathophysiological mechanisms that determine its behavior and the clinical interpretation of this phenomenon. Finally, further studies are needed, aiming to prospectively investigate the prognostic value of the RER overshoot parameters on hard clinical endpoints.

## 5. Conclusions

The present study highlights the role of functional assessment in patients with CHD. An overshoot of RER during recovery after maximal CPET is commonly observed in young patients with CHD but this phenomenon appears to be lower compared to healthy controls, suggesting a possible connection with cardiogenic limitations during exercise. Indeed, RER mag was different in the study’s subgroups where healthy subjects and patients with aortic coarctation showed a significantly higher RER overshoot compared to patients with TGA, previous Fontan procedure, and ToF. RER recovery overshoots correlated with prognostically relevant CPET indices of cardiorespiratory fitness and efficiency, showing lower values in patients with significant ventilatory-perfusion mismatch. The evaluation of CPET recovery parameters should be further investigated and implemented in clinical settings to increase scientific evidence and provide additional information to improve risk stratification in patients with CHD.

## Figures and Tables

**Table 1:** Baseline characteristics of the healthy controls as well as of the patients with congenital heart disease (CHD). Data are expressed as a mean ± the standard deviation. BMI = body mass index; SBP = systolic blood pressure; DBP = diastolic blood pressure; TGA = transposition of great arteries; Fon = Fontan procedure; CoA = aortic coarctation; ToF = tetralogy of Fallot.

Variables	Controls (n = 24)	CHD (n = 93)	TGA (n = 23)	Fon (n = 22)	CoA (n = 24)	ToF (n = 24)
Gender—females, n (%)	11 (46%)	34 (37%)	4 (17%)	9 (41%)	9 (38%)	12 (50%)
Age (years)	14.84 ± 2.69	14.41 ± 3.18	14.39 ± 2.79	14.86 ± 2.92	13.88 ± 3.35	14.54 ± 3.66
Height (cm)	156.00 ± 14.60	161.50 ± 13.51	164.80 ± 10.53	161.82 ± 14.90	159.30 ± 13.80	160.25 ± 14.65
BMI (kg/m^2^)	19.55 ± 2.64	20.70 ± 4.38	22.52 ± 5.23	19.06 ± 2.42	20.45 ± 4.41	20.69 ± 4.46
SBP rest (mmHg)	106.20 ± 15.10	114.25 ± 14.38	118.22 ± 11.92	104.64 ± 15.05	119.92 ± 14.65	113.60 ± 11.50
DBP rest (mmHg)	53.80 ± 9.50	64.92 ± 9.80	63.35 ± 10.30	63.60 ± 8.61	66.12 ± 9.64	66.50 ± 10.65
Desaturation at rest, n (%)	0 (0%)	5 (5%)	0 (0%)	5 (23%)	0 (0%)	0 (0%)
Competitive sports, n (%)	23 (96%)	2 (2%)	0 (0%)	0 (0%)	2 (8%)	0 (0%)
Physical activity (h/week)	5.42 ± 2.67	2.26 ± 1.33	2.14 ± 1.58	2.02 ± 1.32	2.68 ± 1.38	2.12 ± 1.11
Cardio-Aspirine, n (%)	0 (0%)	72 (77%)	1 (4%)	18 (82%)	0 (0%)	2 (8%)
Anti-hypertensive drugs, n (%)	0 (0%)	9 (10%)	1 (4%)	7 (32%)	1 (4%)	0 (0%)

**Table 2:** Cardiopulmonary test parameters of the healthy controls as well as of the patients with congenital heart disease (CHD). Normally distributed variables are expressed with mean ± standard deviation, non-normally distributed variables are expressed with median and confidence intervals; qualitative variables are expressed with percentages of the total. Intergroup comparison was made between the four CHD subgroups and controls. HR = heart rate; HRRec 1 = heart rate recovery after one minute; VO_2_ = oxygen uptake; SBP = systolic blood pressure; DBP = diastolic blood pressure; SpO_2_ = peripheral oxygen saturation; VE/VCO_2_ slope = minute ventilation/carbon dioxide production slope; VT = Ventilatory Threshold; RCP = Respiratory Compensation Point; OUES = Oxygen Uptake Efficiency Slope; METs = metabolic equivalents of task; LVEF = left ventricular ejection fraction; TAPSE = tricuspid annular plane systolic excursion; FAC = fractional area change; * = 86 patients; ** = 64 patients; *** = 22 patients; TGA = transposition of great arteries; Fon = Fontan procedure; CoA = aortic coarctation; ToF = tetralogy of Fallot.

Variables	Controls (n = 24)	CHD (n = 93)	*p*	TGA (n = 23)	Fon (n = 22)	CoA (n = 24)	ToF (n = 24)	*p*
HR peak (bpm)	190.00 (185.35–192.89)	184.00 (178.21–185.41)	0.013	187.00 (173.36–193.25)	176.00 (166.44–180.10)	190.00 (184.74–194.51)	182.50 (174.01–186.74)	<0.001
HR peak (%)	91.00 (89.82–93.70)	90.00 (86.41–89.97)	0.042	92.00 (84.13–94.05)	85.50 (80.80–87.65)	92.00 (89.37–94.22)	89.00 (84.22–90.53)	<0.001
HRRec 1 (-bpm)	−33.00 (−44.12/−30.84)	−26.00 (−31.03/−25.71)	0.007	−28.00 (−35.61–(−24.13))	−18.50 (−25.79–(−16.39))	−30.00 (−37.50–(−27.34))	−26.00 (−35.20–(−23.88))	0.001
HR/VO_2_ slope (bpm/mL)	7.03 ± 3.12	8.34 ± 3.34	0.140	7.30 ± 2.65	9.27 ± 3.72	7.28 ± 2.42	9.55 ± 3.87	0.048
O_2_ pulse (mL/bpm)	11.10 (10.45- 13.55)	10.20 (10.25–11.69)	0.174	11.10 (11.27–13.77)	8.85 (8.44–10.76)	10.85 (9.61–13.07)	9.05 (8.83–11.92)	0.004
O_2_ pulse (%)	104.00 (100.93–118.19)	93.00 (91.02–99.63)	0.005	100.00 (90.19–109.38)	86.00 (76.20–96.98)	97.50 (92.38–108.70)	92.00 (86.76–100.91)	0.004
Oxygen Pulse Behaviour	Normal: 24 (100%)	Normal: 65 (70%) Early Plateau: 25 (27%) Deflection: 3 (3%)	0.007	Normal: 15 (65%) Early Plateau: 6 (26%) Deflection: 2 (9%)	Normal: 16 (73%) Early Plateau: 6 (27%) Deflection: 0	Normal: 21 (87%) Early Plateau: 3 (12%) Deflection: 0	Normal: 13 (54%) Early Plateau: 10 (42%) Deflection: 1 (4%)	0.147
SBP peak (mmHg)	150.00 (140.60–157.00)	150.00 (143.9–153.40)	0.947	150.00 (142.77–157.66)	137.50 (130.25–147.02)	150.00 (146.54–168.88)	147.50 (136.69–157.89)	0.152
DBP peak (mmHg)	50.00 (45.48–56.12)	60.00 (58.29–64.40)	<0.001	60.00 (55.90–69.75)	60.00 (54.24–65.77)	50.00 (52.37–65.55)	60.00 (57.27–69.81)	0.012
SpO_2_ peak (%)	99.00 (98.86–99.53)	96.00 (95.11–96.84)	<0.001	98.00 (97.37–98.36)	92.00 (89.46–94.14)	98.00 (97.03–98.88)	97.50 (93.85–97.42)	<0.001
Desaturation at peak, n (%)	0 (0%)	19 (21%)	0.012	0 (0%)	12 (60%)	1 (4%)	6 (27%)	<0.001
VE/VCO_2_ slope	27.93 (26.36–29.35)	29.14 (28.73–30.61)	0.102	28.25 (27.14–30.72)	31.06 (29.10–33.20)	28.17 (26.47–30.47)	28.68 (28.28–32.17)	0.120
OUES (mL/logL)	1849.19 (1739.05–2281.50)	1784.00 (1739.96–1994.13)	0.365	1990.00 (1804.69–2220.96)	1613.50 (1460.38–1949.52)	1887.50 (1695.67–2342.09)	1565.50 (1475.68–1972.49)	0.087
VO_2_ peak (mL/min/kg)	43.72 ± 6.13	36.27 ± 8.33	<0.001	36.83 ± 8.70	32.05 ± 5.90	40.98 ± 8.40	34.90 ± 7.85	<0.001
VO_2_ peak (%)	108.84 ± 15.82	86.70 ± 17.90	<0.001	83.30 ± 15.80	76.64 ± 14.40	99.00 ± 17.10	86.92 ± 17.10	<0.001
VO_2_ at VT (mL/Kg/min)	23.90 (22.34–26.22)	22.80 (21.81–24.50)	0.229	22.80 (21.11–24.22)	21.40 (19.47–22.89)	25.60 (22.82–26.26)	21.60 (19.19–29.06)	0.085
VO_2_ at RCP (mL/Kg/min)	34.50 (32.28–38.89)	28.200 (27.69–30.57)	<0.001	28.80 (25.93–31.63)	25.10 (23.52–28.75)	31.45 (29.83–36.15)	28.00 (25.50–30.76)	<0.001
METs	16.76 ± 2.09	15.03 ± 2.48	0.001	15.00 ± 2.61	14.72 ± 1.95	15.65 ± 2.80	14.75 ± 2.52	0.040
LVEF * (%)	-	64.00 (60.00–70.00)	-	63.00 (61.25–69.00)	58.00 (50.47–63.24)	68.00 (64.51–70.93)	66.00 (61.08–68.52)	0.002
TAPSE ** (mm)	-	19.00 (16.40–22.10)	-	17.00 (15.21–18.10)	12.60 (9.15–18.59)	25.10 (22.24–27.10)	19.00 (16.67–20.59)	<0.001
FAC *** (%)	-	44.50 (40.00–48.00)	-	40.50 (34.15–46.85)	48.00 (33.63–55.17)	42.50 (37.60–47.40)	44.00 (38.89–46.66)	0.392

**Table 3:** Respiratory exchange ratio (RER) recovery parameters of the healthy controls as well as of the patients with congenital heart disease (CHD). Normally distributed variables are expressed with mean ± standard deviation and non-normally distributed variables are expressed with median and confidence intervals. Intergroup comparison was made between the four CHD subgroups and controls. TGA = transposition of great arteries; Fon = Fontan procedure; CoA = aortic coarctation; ToF = tetralogy of Fallot.

Variables	Controls (n = 24)	CHD (n = 93)	*p*	TGA (n = 23)	Fon (n = 22)	CoA (n = 24)	ToF (n = 24)	*p*
RER peak	1.22 ± 0.11	1.23 ± 0.12	0.819	1.24 ± 0.14	1.22 ± 0.11	1.20 ± 0.12	1.24 ± 0.10	0.902
RER max	1.94 ± 0.28	1.77 ± 0.23	0.010	1.80 ± 0.25	1.74 ± 0.24	1.80 ± 0.21	1.75 ± 0.24	0.076
RER mag (%)	58.54 ± 14.72	44.41 ± 14.75	0.010	43.74 ± 13.81	42.31 ± 13.10	49.95 ± 15.23	41.42 ± 15.94	0.001
RER slope	34.40 ± 16.50	27.63 ± 13.52	0.037	29.81 ± 15.40	23.12 ± 10.84	28.80 ± 13.70	28.50 ± 13.60	0.207
Time to RER max (s)	146.00 (126.31–164.17)	139.00 (129.47–151.50)	0.403	130.00 (107.60–151.87)	151.50 (137.58–186.60)	145.00 (119.90–170.51)	119.00 (108.58–143.92)	0.136

**Table 4:** Correlations between the respiratory exchange ratio (RER) recovery parameters and cardiopulmonary/echocardiographic functional indices, expressed as Pearson’s or Spearman’s indices.

Variables	RER Peak	RER Max	RER Mag	RER Slope	Time to RER Max
Age (years)	0.428 (*p* = 0.001)	0.277 (*p* = 0.007)	−0.034 (*p* = 0.744)	−0.085 (*p* = 0.418)	0.174 (*p* = 0.095)
HR peak (bpm)	0.227 (*p* = 0.021)	0.323 (*p* = 0.001)	0.366 (*p* = 0.001)	0.297 (*p* = 0.004)	−0.042 (*p* = 0.692)
HR/VO_2_ slope (bpm/mL)	0.059 (*p* = 0.581)	−0.135 (*p* = 0.103)	−0.232 (*p* = 0.004)	−0.154 (*p* = 0.418)	−0.095 (*p* = 0.089)
HRRec 1 (-bpm)	0.412 (*p* < 0.001)	0.253 (*p* = 0.014)	−0.042 (*p* = 0.687)	0.122 (*p* = 0.242)	0.006 (*p* = 0.950)
VE/VCO_2_ slope	−0.429 (*p* = 0.001)	−0.343 (*p* = 0.001)	−0.100 (*p* = 0.334)	−0.201 (*p* = 0.054)	−0.021 (*p* = 0.840)
VO_2_ at VT (ml/kg/min)	−0.265 (*p* = 0.008)	−0.115 (*p* = 0.274)	0.100 (*p* = 0.343)	0.123 (*p* = 0.245)	−0.060 (*p* = 0.573)
VO_2_ peak (ml/kg/min)	−0.100 (*p* = 0.314)	0.212 (*p* = 0.040)	0.393 (*p* = 0.001)	0.297 (*p* = 0.004)	−0.048 (*p* = 0.650)
OUES (ml/logL)	0.213 (*p* = 0.031)	0.370 (*p* = 0.001)	0.311 (*p* = 0.002)	0.137 (*p* = 0.191)	0.143 (*p* = 0.170)
LVEF (%)	0.056 (*p* = 0.608)	−0.003 (*p* = 0.975)	0.002 (*p* = 0.989)	0.083 (*p* = 0.449)	−0.072 (*p* = 0.510)
TAPSE (mm)	0.241 (*p* = 0.057)	0.312 (*p* = 0.012)	0.151 (*p* = 0.234)	−0.083 (*p* = 0.516)	0.192 (*p* = 0.132)
FAC (%)	0.199 (*p* = 0.365)	−0.068 (*p* = 0.764)	−0.065 (*p* = 0.772)	0.035 (*p* = 0.877)	−0.090 (*p* = 0.692)

## Data Availability

Data are available upon reasonable request to the corresponding author.

## Supplementary Materials

The following supporting information can be downloaded at: https://www.mdpi.com/article/10.3390/children10030521/s1, Table S1. Pairwise comparisons between four CHD groups and controls.
