# Supplementary material for: Overshoot of the Respiratory Exchange Ratio during Recovery from Maximal Exercise Testing in Young Patients with Congenital Heart Disease
Source: Children (Basel). 2023 Mar 7;10(3):521. doi: 10.3390/children10030521 (PMC10047014; doi:10.3390/children10030521)
Supplement: Supplementary file 1 [file children-10-00521-s001.zip › children-2167480-supplementary.pdf]

Supplementary Table S1. Pairwise comparisons between four CHD groups and controls.

**HR peak (bpm)**	**p**
Fon vs ToF	0.052
Fon vs TGA	0.001
Fon vs controls	0.000
Fon vs CoA	0.000
ToF vs TGA	0.170
ToF vs controls	0.027
ToF vs CoA	0.021
TGA vs controls	0.427
TGA vs CoA	0.365
controls vs CoA	0.904

**HR peak (%)**	**p**
Fon vs ToF	0.105
Fon vs TGA	0.002
Fon vs controls	0.000
Fon vs CoA	0.000
ToF vs TGA	0.115
ToF vs controls	0.029
ToF vs CoA	0.026
TGA vs controls	0.569
TGA vs CoA	0.532
controls vs CoA	0.951

**HRRec 1 (- bpm)**	p
controls vs CoA	0.374
controls vs TGA	0.094
controls vs ToF	0.059
controls vs Fon	0.000
CoA vs TGA	0.431
CoA vs ToF	0.323
CoA vs Fon	0.001
TGA vs ToF	0.850
TGA vs Fon	0.017
ToF vs Fon	0.026

**SpO_2 peak (%)**	p
Fon vs ToF	0.034
Fon vs TGA	0.000
Fon vs CoA	0.000
Fon vs controls	0.000
ToF vs TGA	0.153
ToF vs CoA	0.033
ToF vs controls	0.000
TGA vs CoA	0.484
TGA vs controls	0.002
CoA vs controls	0.017

**O_2 pulse (mL/bpm)**	p
Fon vs ToF	0.449
Fon vs CoA	0.070
Fon vs controls	0.011
Fon vs TGA	0.001
ToF vs CoA	0.280
ToF vs controls	0.068
ToF vs TGA	0.009
CoA vs controls	0.463
CoA vs TGA	0.126
controls vs TGA	0.412

**O_2 pulse (%)**	p
Fon vs ToF	0.133
Fon vs TGA	0.024
Fon vs CoA	0.016
Fon vs controls	0.000
ToF vs TGA	0.426
ToF vs CoA	0.348
ToF vs controls	0.022
TGA vs CoA	0.894
TGA vs controls	0.144
CoA vs controls	0.179

**DBP peak (mmHg)**	p
controls vs CoA	0.052
controls vs Fon	0.020
controls vs TGA	0.004
controls vs ToF	0.001
CoA vs Fon	0.675
CoA vs TGA	0.340
CoA vs ToF	0.207
Fon vs TGA	0.604
Fon vs ToF	0.415
TGA vs ToF	0.768

**HR/VO_2 slope (bpm/ml)**	p
controls vs TGA	0.501
controls vs CoA	0.312
controls vs ToF	0.011
controls vs Fon	0.020
TGA vs CoA	0.747
TGA vs ToF	0.068
TGA vs Fon	0.092
CoA vs ToF	0.128
CoA vs Fon	0.159
ToF vs Fon	0.962

**METs**	**p**
Fon vs ToF	0.792
Fon vs TGA	0.386
Fon vs CoA	0.280
Fon vs controls	0.005
ToF vs TGA	0.535
ToF vs CoA	0.402
ToF vs controls	0.009
TGA vs CoA	0.829
TGA vs controls	0.052
CoA vs controls	0.084

**VO_2 peak (mL/min/kg)**	p
Fon vs ToF	0.133
Fon vs TGA	0.024
Fon vs CoA	0.000
Fon vs controls	0.000
ToF vs TGA	0.427
ToF vs CoA	0.029
ToF vs controls	0.000
TGA vs CoA	0.173
TGA vs controls	0.005
CoA vs controls	0.149

**LVEF**	p
Fon vs ToF	0.008
Fon vs TGA	0.017
Fon vs controls	-
Fon vs CoA	0.001
ToF vs TGA	0.806
ToF vs controls	-
ToF vs CoA	0.226
TGA vs controls	-
TGA vs CoA	0.152
CoA vs controls	-

**VO_2 peak (%)**	p
Fon vs TGA	0.245
Fon vs ToF	0.044
Fon vs CoA	0.000
Fon vs controls	0.000
TGA vs ToF	0.394
TGA vs CoA	0.003
TGA vs controls	0.000
ToF vs CoA	0.032
ToF vs controls	0.000
CoA vs controls	0.105

**TAPSE**	p
Fon vs ToF	0.754
Fon vs TGA	0.678
Fon vs controls	-
Fon vs CoA	0.048
ToF vs TGA	0.141
ToF vs controls	-
ToF vs CoA	0.001
TGA vs controls	-
TGA vs CoA	0.000
CoA vs controls	-

**VO_2 at RCP (mL/Kg/min)**	p
Fon vs ToF	0.144
Fon vs TGA	0.077
Fon vs CoA	0.001
Fon vs controls	0.000
ToF vs TGA	0.771
ToF vs CoA	0.048
ToF vs controls	0.002
TGA vs CoA	0.089
TGA vs controls	0.005
CoA vs controls	0.257

**RER mag (%)**	p
ToF vs Fon	0.981
ToF vs TGA	0.649
ToF vs CoA	0.102
ToF vs controls	0.000
Fon vs TGA	0.674
Fon vs CoA	0.115
Fon vs controls	0.001
TGA vs CoA	0.244
TGA vs controls	0.002
CoA vs controls	0.058

HR = heart rate; HRRec 1 = heart rate recovery after one minute; VO_2 = oxygen uptake; DBP = diastolic blood pressure; SpO_2 = peripheral oxygen saturation; RCP = respiratory compensation point; METs = metabolic equivalents of task; LVEF = left ventricular ejection fraction; TAPSE = tricuspid annular plane systolic excursion; RER mag = respiratory exchange ratio magnitude; TGA = transposition of great arteries; Fon = Fontan procedure; CoA = aortic coarctation; ToF = tetralogy of Fallot.
